# Growth Dynamics and Toxin Production of *Pseudo-nitzschia* Species Isolated from the Central Adriatic Sea

**DOI:** 10.3390/toxins17060307

**Published:** 2025-06-17

**Authors:** Tina Tomašević, Jasna Arapov, Ivana Ujević, Tina Bonačić, Mia Bužančić, Antonija Bulić, Sanda Skejić, Romana Roje-Busatto, Živana Ninčević Gladan

**Affiliations:** 1 Institute of Oceanography and Fisheries, Šetalište Ivana Meštrovića 63, 21000 Split, Croatia; arapov@izor.hr (J.A.); ujevic@izor.hr (I.U.); bonacic@izor.hr (T.B.); buzancic@izor.hr (M.B.); bulic@izor.hr (A.B.); sanda@izor.hr (S.S.); rroje@izor.hr (R.R.-B.); nincevic@izor.hr (Ž.N.G.); 2 Doctoral Study of Biophysics, Faculty of Science, University of Split, Ruđera Boškovića 33, 21000 Split, Croatia

**Keywords:** Adriatic Sea, domoic acid, growth rate, toxic diatoms

## Abstract

The marine diatoms *Pseudo-nitzschia* spp. are globally distributed primary producers, with certain species capable of producing neurotoxin domoic acid (DA), causing amnesic shellfish poisoning (ASP). This study investigates the toxicity and growth rates of *Pseudo-nitzschia* species isolated from aquaculture areas in the Central Adriatic Sea. A total of 54 strains from eight species were analysed, with strains originating from four different study sites. Growth rates and toxin production were examined across different growth phases in other species and strains. Most species, including *P. allochrona*, *P. calliantha*, *P. delicatissima*, *P. fraudulenta*, *P. galaxiae*, *P. mannii*, and *P. multistriata*, did not produce DA at any growth phase. The only species that showed toxin production, at 18 °C was *P. pseudodelicatissima* (0.0007–0.0250 pg cell^−1^). Notably, the majority of *P. pseudodelicatissima* strains were toxic in the decay phase and some strains showed continuous toxin production throughout all growth phases. The highest growth rates for the analysed species were recorded in strains of *P. delicatissima,* which also exhibited the highest cell abundance (8.19 × 10^5^ cell mL^−1^), followed by the species *P. allochrona*, *P. mannii* and *P. pseudodelicatissima.*

## 1. Introduction

The marine diatoms *Pseudo-nitzschia* spp. are globally distributed primary producers [1]. Of the 63 species of the genus *Pseudo-nitzschia*, 28 have been identified as producers of the neurotoxin domoic acid (DA) [2,3,4,5,6]. Since DA is an analogue of glutamate, it causes neurological complications such as disorientation and memory loss, which is why DA poisoning is also referred to as amnesic shellfish poisoning (ASP) [7]. Domoic acid enters the marine food web and accumulates in shellfish and filter-feeding fish, which can lead to ASP when humans and various marine animals (e.g., seabirds, sea lions, cephalopods) consume contaminated filter-feeding organisms [8]. Further studies have shown that DA causes neurological symptoms even in filter-feeding fish, such as anchovies [9]. However, DA does not exert the same effect on shellfish, mainly because their nervous systems are less developed than those of birds and mammals, which could explain differences in variations in susceptibility to DA [1]. The first reported ASP event occurred in 1987 on Prince Edward Island, Canada, when three people died and at least 107 illnesses [10,11] were caused by intoxication. The *Pseudo-nitzschia* species that caused the poisoning was *Pseudo-nitzschia multiseries*, and DA levels of up to 790 µg g^−1^ were detected in blue mussels (*Mytilus galloprovincialis*) [12,13].

In the Adriatic Sea, DA was first detected in 2000 in blue mussel (*Mytilus galloprovincialis*) tissue collected from the Italian West coast [14]. Domoic acid was detected at low concentrations (2.5 μg g^−1^); however, toxin monitoring has since been initiated to ensure the safety of mussels for consumption. According to Regulation (EC) 853/2004 [15] and Croatian legislation, the maximum permitted level of DA in edible shellfish tissue is 20 µg g^−1^. Over the years, DA has been detected in shellfish from aquaculture areas; however, the concentrations have remained below the regulatory limit. The first reported occurrence of DA in Croatian waters was recorded in January 2006, detected in the blue mussel (*M. galloprovincialis*) with a maximum concentration of 6.5486 μg g^−1^ [16]. Since then, DA has occasionally been detected in mussels from aquaculture areas at low concentrations, consistently below the regulatory limit. Its presence has been confirmed in the Northern Adriatic Sea with DA concentrations in shellfish tissue ranging from 0.097 to 1.320 μg g^−1^ [17,18,19], and DA has also been detected in the Central Adriatic Sea with concentrations ranging from 0.17 to 3.24 μg g^−1^ [20,21,22].

Species of the genus *Pseudo-nitzschia* are commonly present in the Adriatic Sea phytoplankton community [16,23], and with occasional blooms occurring in all seasons [16,18,24,25]. To date, 14 species of the genus *Pseudo-nitzschia* have been described in the Adriatic Sea [17,24,26,27,28,29,30]. The production of DA has been identified in five species: *P. delicatissima* [31,32,33], *P. calliantha* [32,34], *P. multistriata* [33], *P. galaxiae* [33], and *P. pseudodelicatissima* [32], with most studies conducted in the Northern Adriatic region.

Accordingly, this study aims to investigate the following: (i) the toxicity of various *Pseudo-nitzschia* species isolated from field populations in aquaculture areas of the Central Adriatic Sea; (ii) the variation in toxin production across different strains of each species and various growth phases; (iii) the difference in growth rates for each analysed *Pseudo-nitzschia* strain.

## 2. Results

**Figure 1:**
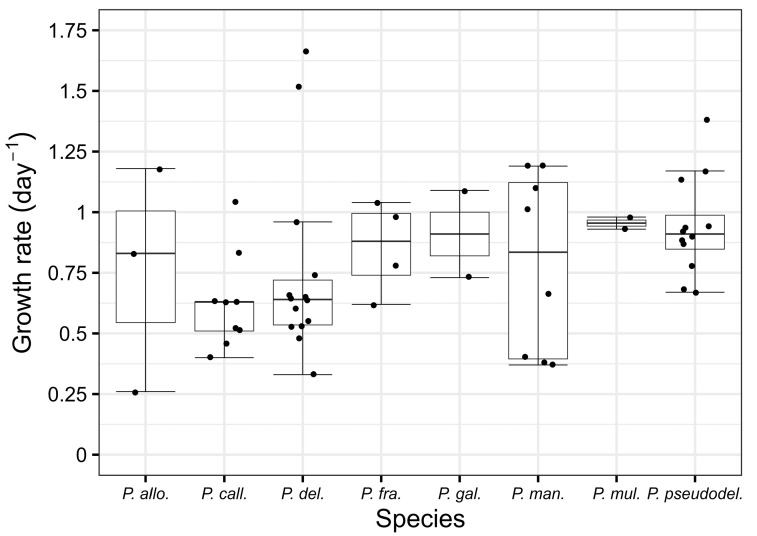
Growth rates of all analysed strains for each species. The centre line represents the median value, the lower edge of the box the lower quartile; the upper edge of the box the upper quartile. The T-shaped whiskers go to the last point, which is still within 1.5 times the interquartile range. If there are no outliers, the whiskers present the maximum or minimum values.

In this study, toxin production and growth rates were analysed for 54 strains of the genus *Pseudo-nitzschia* spp., comprising a total of eight species: *P. allochrona*, *P. calliantha*, *P. delicatissima*, *P. fraudulenta*, *P. galaxiae*, *P. mannii*, *P. multistriata*, and *P. pseudodelicatissima* (Figure 1, Table 1). Three different species were isolated from the Velebit Channel study site (*P. calliantha*, *P. delicatissima*, *P. mannii*), while two different species were isolated from the Šibenik Channel (*P. allochrona* and *P. multistriata*). Five different species were isolated from Kaštela Bay (*P. delicatissima*, *P. fraudulenta*, *P. galaxiae*, *P. mannii*, and *P. pseudodelicatissima*), and four species from Mali Ston Bay (*P. calliantha*, *P. delicatissima*, *P. galaxiae*, and *P. mannii*).

### 2.1. Pseudo-nitzschia allochrona

**Figure 2:**
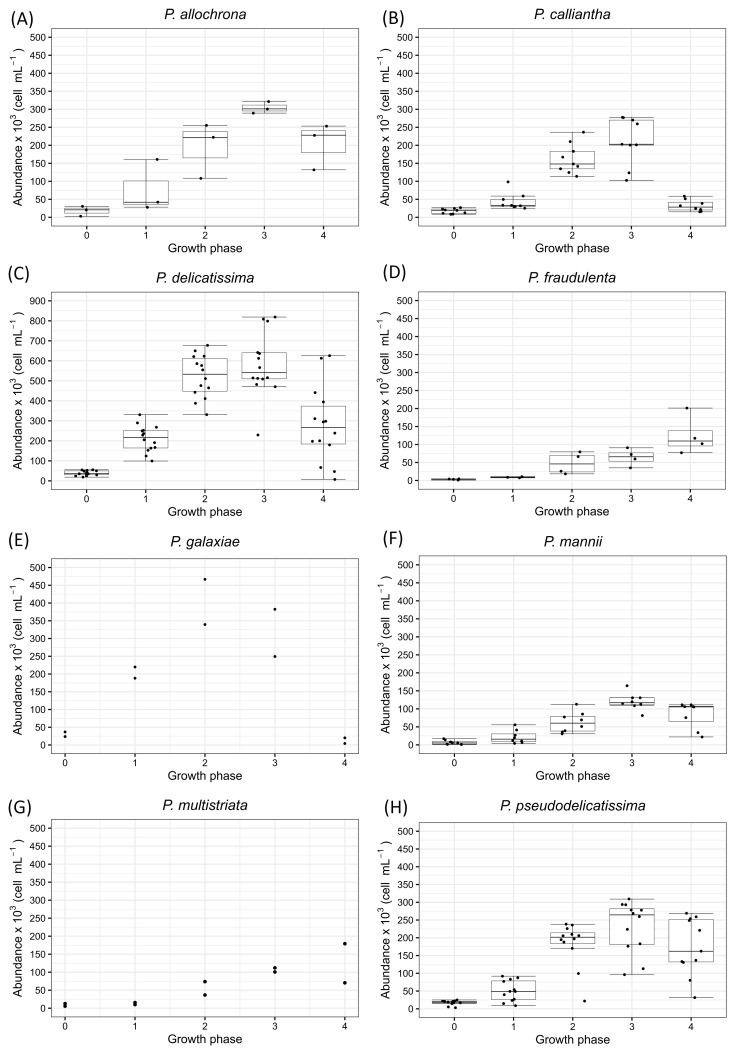
Cell abundance (cell mL^−1^) by growth phase for all analysed strains of *Pseudo-nitzschia* species. (**A**) *P. allochrona*; (**B**) *P. calliantha*; (**C**) *P. delicatissima*; (**D**) *P. fraudulenta*; (**E**) *P. galaxiae*; (**F**) *P. mannii*; (**G**) *P. multistriata*; (**H**) *P. pseudodelicatissima*. The scale is different for the *P. delicatissima* due to high cell abundance. The centre line represents the median value, the lower edge of the box the lower quartile; the upper edge of the box the upper quartile. The T-shaped whiskers go to the last point, which is still within 1.5 times the interquartile range. If there are no outliers, the whiskers present the maximum or minimum values.

Three strains of *P. allochrona* were analysed for growth rate and toxicity. The strains were isolated in late summer/early fall from one study site, the Šibenik Channel (strains S222al, S223al, and S280al). Temperature during *P. allochrona* strains analysis was 19 °C. The initial cell abundance was between 2.64 × 10^3^ and 30.34 × 10^3^ cells mL^−1^ (Figure 2A). The maximum cell abundance ranged from 289.40 × 10^3^ to 322.20 × 10^3^ cells mL^−1^ and was observed in the stationary phase (growth phase 3). For the *P. allochrona* strains, the growth rate interval ranged from 0.26–1.18 day^−1^. The cell abundances on the day of inoculation exhibited minimal deviations, while the abundances in the late exponential phase (growth phase 2) showed the greatest fluctuations. All three *P. allochrona* strains did not produce the DA toxin, regardless of the growth phase of the cell culture.

### 2.2. Pseudo-nitzschia calliantha

A total of nine strains of *P. calliantha* were examined for growth rate and toxicity, isolated from two study sites: Velebit Channel (strains V061ca, V065ca, V068ca, V070ca, V071ca, V072ca, V077ca, and V079ca) and Mali Ston Bay (strain M074ca). All *P. calliantha* strains were isolated in winter and cultivated at 18 °C. The initial cell abundance ranged from 8.49 × 10^3^ to 24.60 × 10^3^ cells mL^−1^, while the maximum cell abundance ranged from 113.75 × 10^3^ to 277.60 × 10^3^ cells mL^−1^. The highest cell abundances were recorded in growth phase 3 (Figure 2B). The interval of the growth rate was 0.40–1.04 day^−1^. The smallest difference in cell abundance between the strains of *P. calliantha* was observed on the day of inoculation, while the largest difference in cell abundance was observed in growth phase 3. The tested strains of *P. calliantha* did not produce the toxin DA regardless of the growth phase of the cell culture.

### 2.3. Pseudo-nitzschia delicatissima

Fourteen analysed strains of *P. delicatissima* were isolated in the fall and late winter from the three study sites: Kaštela Bay (strains K057de, K058de, K129de and K134de), Velebit Channel (strains V040de, V041de, V042de and V043de) and Mali Ston Bay (strains M075de, M085de, M088de, M144de, M461de and M462de). The *P. delicatissima* strains were analysed under temperature conditions ranging from 16 to 18 °C. Cell abundances on the day of inoculation were between 18.78 × 10^3^ and 55.28 × 10^3^ cells mL^−1^ (Figure 2C). The highest cell abundances were recorded in the stationary phase (growth phase 3), ranging from 411.20 × 10^3^ to 819.00 × 10^3^ cells mL^−1^. The interval of the growth rate was 0.33–1.66 day^−1^. The maximum difference between cell abundances was observed in the decay phase of the cell culture (growth phase 4). The tested strains of *P. delicatissima* did not produce the toxin DA regardless of the growth phase of the cell culture.

### 2.4. Pseudo-nitzschia fraudulenta

Four strains of *P. fraudulenta* were analysed; all four strains were isolated from Kaštela Bay at the beginning of the winter (strains K450fr, K455fr, K456fr, and K458fr). The analysis of *P. fraudulenta* cell cultures was performed at 16 °C. These strains were inoculated with an initial cell count of 1.04 × 10^3^ to 4.51 × 10^3^ cells mL^−1^ (Figure 2D). The maximum cell abundances were counted in the growth phase 4 in the range of 77.10 × 10^3^–201.30 × 10^3^ cells mL^−1^. The interval of the growth rate was between 0.62 and 1.04 day^−1^. The minimum difference between the cell abundances among strains was noticed in the early exponential phase (growth phase 1), while the maximum difference between cell abundances among strains was found in growth phase 4, which in this case was not the decay phase. In this strain, growth phase 4 could be described as the late stationary phase. The investigated strains of *P. fraudulenta* did not produce DA.

### 2.5. Pseudo-nitzschia galaxiae

Two *P. galaxiae* isolates were analysed: one from Kaštela Bay (strain K136ga) analysed at 18 °C and one from Mali Ston Bay (strain K232ga) analysed at 19 °C. The initial cell abundances were 4.28 × 10^3^ and 36.76 × 10^3^ cells mL^−1^, while the maximum cell abundances were 382.40 × 10^3^ and 466.80 × 10^3^ cells mL^−1^, recorded in growth phase 3 (Figure 2E). The growth rates were 0.73 and 1.09 day^−1^, respectively. Two examined strains of *P. galaxiae* did not show production of toxin DA regardless of the growth phase of the cell culture.

### 2.6. Pseudo-nitzschia mannii

Eight *P. mannii* strains were analysed for toxin production and growth rate, isolated in late summer from three study sites: Velebit Channel (strain V229ma), Mali Ston Bay (strains M233ma, M236ma, M239ma, M240ma, and M241ma), and Kaštela Bay (strains K231ma and K237ma). All *P. mannii* strains were analysed at 19 °C. On the day of inoculation, the minimum cell abundance in these *P. mannii* strains was 930 cells mL^−1^, while the maximum cell abundance on the day of inoculation was 17.10 × 10^3^ cells mL^−1^ (Figure 2F). The highest cell abundances were recorded in growth phase 3 (stationary phase) and ranged from 108.70 × 10^3^ to 164.35 × 10^3^ cells mL^−1^. The calculated growth rates ranged from 0.37 to 1.19 day^−1^. The smallest differences in cell abundances were observed on the day of inoculation, while the largest differences occurred during the decay phase of the cell culture (growth phase 4). Of the eight isolated strains of *P. mannii*, none exhibited toxin production.

### 2.7. Pseudo-nitzschia mutistriata

Two strains of *P. multistriata* isolated from the Šibenik Channel in fall (strain S290mu and S442mu) were analysed. The strain S290 was analysed at 18 °C, while strain S442 was analysed at 16 °C. The strains were inoculated with an initial cell count of 5.67 × 10^3^ and 12.77 × 10^3^ cells mL^−1^ (Figure 2G). For these two strains, the maximum cell count in cell culture was 100.60 × 10^3^ cells mL^−1^ and 179.20 × 10^3^ cells mL^−1^. For strain S290mu, the maximum cell number was recorded in growth phase 4; however, this strain did not show any decay phase, so that growth phase 4, in this case, represents the stationary phase. For strain S442mu, the maximum cell number was recorded in growth phase 3. The growth rates were 0.93 and 0.98 day^−1^, respectively. These *P. multistriata* strains showed no toxin production.

### 2.8. Pseudo-nitzschia pseudodelicatissima

**Figure 3:**
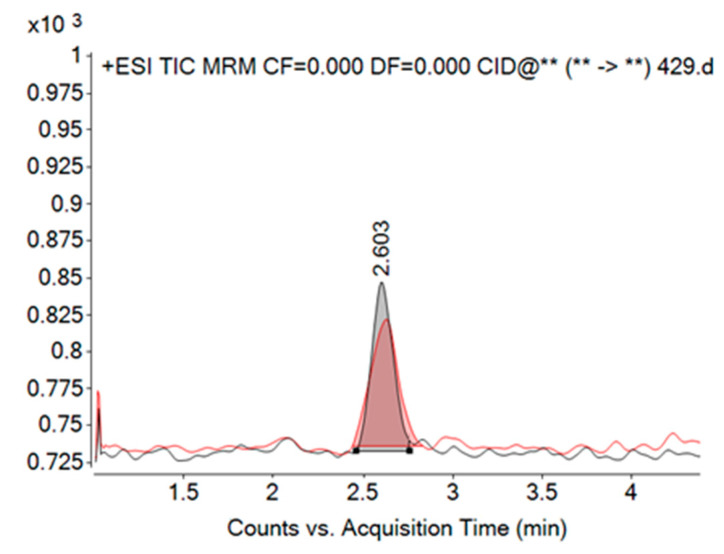
LC–MS/MS chromatogram obtained by LC–MS/MS analysis of the *Pseudo-nitzschia pseudodelicatissima* strain K357ps at the stationary growth phase (growth phase 3), containing a domoic acid peak at retention time 2.603 min (black trace). The red trace represents the domoic acid standard included for comparison.

**Figure 4:**
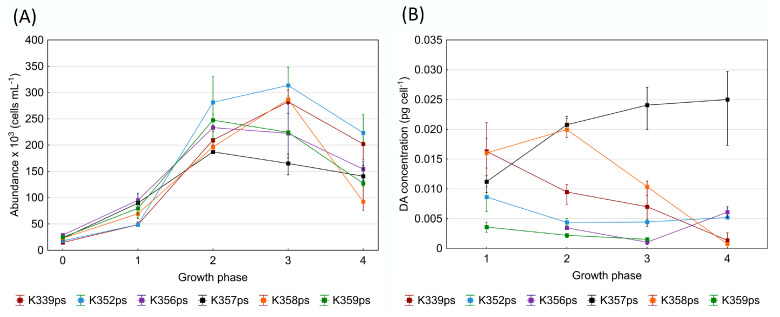
(**A**) Cell abundance (cell mL^−1^) through growth phases of strains showing toxin production in at least three growth phases. (**B**) Mean domoic acid concentration (pg cell^−1^) in replicate samples during the growth phases of strains that produced the toxin in at least three distinct phases.

In this study, twelve strains of *P. pseudodelicatissima* were analysed; all the strains were isolated from the Kaštela Bay study site in late fall. All *P. pseudodelicatissima* cell cultures were analysed for toxicity at a constant temperature of 18 °C throughout the cultivation period. The studied strains were inoculated with initial cell counts between 2.75 × 10^3^ and 25.18 × 10^3^ cells mL^−1^ (Figure 2H), while the maximum counted cell abundance ranged from 96.27 × 10^3^ to 309.20 × 10^3^ cells mL^−1^. The highest cell abundances were recorded for all strains in growth phase 3. The mean growth rate interval varied from 0.60 to 1.46 day^−1^. Of the strains examined, 11 exhibited toxin production (Figure 3), with strain K350ps being the only one that did not produce toxin at any growth phase. Continuously high toxin production was observed in strain K357ps, which displayed the highest toxin concentration among all toxic strains (0.0250 pg cell^−1^), measured during the decay phase (growth phase 4) (Table 2). Similarly, strain K358ps showed high toxin production (0.0200 pg cell^−1^), with the highest concentration occurring during the late exponential growth phase (growth phase 2). In the majority of the strains examined, toxin production was observed during the decay phase (growth phase 4), except for K328ps and K359ps. Four strains (K339ps, K352ps, K357ps, and K358ps) exhibited toxin production consistently across all four analysed growth phases. In these strains, which exhibited continuous toxin production, the toxin content of the cells decreased throughout the growth cycle, with the highest toxin concentration recorded in the early exponential phase (growth phase 1) (Figure 4). The only exception was strain K357ps, in which the toxin concentration gradually increased during the growth phases and reached its peak in the decay phase (growth phase 4).

## 3. Discussion

### 3.1. Abundance and Growth Rate

This research represents the first comprehensive study of the growth rate and toxicity data during a continuous cell culture experiment that included 54 strains of the genus *Pseudo-nitzschia*. The study includes eight species isolated from the Central Adriatic Sea: *P. allochrona*, *P. calliantha*, *P. delicatissima*, *P. fraudulenta*, *P. galaxiae*, *P. mannii*, *P. multistriata*, and *P. pseudodelicatissima*. Of the species examined, strains of *P. delicatissima* exhibited the highest cell abundances during the entire growth cycle, up to 819.00 × 103 cell mL^−1^, which was also observed in previous studies, in which strains of *P. delicatissima* reached cell counts up to 1.6 × 10^6^ cell mL^−1^ [35]. Similarly, high cell counts were determined in this study for *P. galaxiae*, with values of up to 466.80 × 10^3^ cells mL^−1^. This species is also known to reach high abundances in natural environments [33,36,37]. In this study, the *P. calliantha* strains reached maximum cell abundances ranging from 113.75 × 10^3^ to 277.60 × 10^3^ cells mL^−1^. In comparison, previous studies reported maximum abundances for *P. calliantha* in cell culture ranged between 272 × 10^3^ and 1.97 × 10^6^ cells mL^−1^ [38]. Regarding the obtained growth rates, the highest values among the species studied were recorded for *P. delicatissima*, ranging from 1.52 to 1.66 day^−1^, followed by *P. pseudodelicatissima* (1.13 to 1.38 day^−1^) and *P. mannii* (1.10 to 1.19 day^−1^). Notably, it was observed that in some cases, initial inoculation with high cell densities resulted in lower growth rates, but the exponential growth phase was still recognizable. Furthermore, in several strains (S290mu, K450fr, K455fr, K456fr, and K458fr), the decay phases were not recognizable, and the growth phases 4 were the stationary phases. A possible explanation for these discrepancies could be slight temporal sampling issues that may have affected the interpretation of growth dynamics, although the approach was generally effective. In addition, this study confirmed that several factors influence cell abundance, growth rate and logistic growth, including the growth phase of the batch/original cell culture at the time of inoculation, counting errors and sample preparation techniques. These results are consistent with those of Turk Dermastia et al. [33] and support the complexity of factors determining the growth dynamics of *Pseudo-nitzschia* species.

### 3.2. Toxicity

All the analysed strains were monitored through four growth phases for DA production. Of the eight *Pseudo-nitzschia* species analysed in this study, only *P. pseudodelicatissima*, growing at 18 °C, produced detectable levels of domoic acid (DA), with concentrations ranging from 0.0007 to 0.0250 pg cell^−1^. It is noteworthy that the *P. pseudodelicatissima* strains were isolated from field samples collected during a period of elevated DA concentrations in mussels (unpublished data). Given this temporal correlation, it was expected that the isolated *Pseudo-nitzschia* strains would also produce toxins, providing a good basis for further investigation of their toxicity profiles and growth dynamics. Previous studies have reported variable DA production in *P. pseudodelicatissima* strains cultivated at different temperatures. A strain isolated from the Northern Adriatic Sea and grown at 16 °C exhibited lower DA levels, with a maximum concentration of 1.01 fg cell^−1^ [32]. Similarly, Lapworth et al. [39] reported toxin production in only one of five strains isolated from Australian waters and cultivated at 14 °C. In contrast, all six strains from the Thermaikos Gulf (Greece) produced DA when analysed at 19 °C [40], while strains cultivated at higher temperatures, such as 20 °C (Gulf of Naples) and 24 °C (Denmark Strait), did not exhibit DA production [41,42]. These results suggest that DA production in *P. pseudodelicatissima* may be influenced by cultivation temperature, with moderate thermal conditions (ca. 16–19 °C) favoring toxin biosynthesis. In contrast, elevated temperatures (≥20 °C) may suppress DA production. Other potential cofactors, such as nutrient availability, light conditions, and sampling season, may also influence the toxin biosynthesis and act as confounding variables. Species *P. psedudodelicatissima* have not yet been thoroughly investigated under varying environmental conditions, and further research is needed to better understand their roles in toxicity [1].

The analysed strain, K357ps, showed the highest toxin production (0.0250 pg cell^−1^) in the decay phase, with a relatively high cell number, and nine out of eleven strains produced DA during the decay phase, which is consistent with the findings of Moschandreou et al. [40], who also recorded toxin production in strains of *P. pseudodelicatissima* during the decay phase. In contrast, *P. calliantha* isolated from the Central Adriatic Sea confirmed the highest toxin concentrations in the early stage of development (0.0351–0.0855 pg cell^−1^) with the lowest cell abundance of the growth cycle [34]. Similarly, strains of *P. multistriata* from the Northern Adriatic produced toxin in the early growth phase, also with the lowest cell abundance [33]. In this study, different results were observed for DA production in relation to cell density. The *P. pseudodelicatissima* strain K352ps, from this experiment with the lowest cell abundance in the early growth phase, produced DA at a concentration three times lower (0.0086 pg cell^−1^) than the highest DA concentration produced in this experiment. The strain K336ps with the highest cell number in the stationary growth phase had a relatively low toxin concentration (0.0014 pg cell^−1^). It is well established that DA production, depending on cell abundance and growth phase, varies between different species and even among strains within the same species, as observed by Thessen et al., where only two out of nine *P. calliantha* and two out of five *P. fraudulenta* isolates were found to be toxic [38]. Different *Pseudo-nitzschia* species and strains could have different patterns of producing DA and start producing DA during the exponential growth phase when cell division is rapid, while others could have increased DA production during the stationary phase when growth slows down due to nutrient deficiency or other environmental stress factors. In addition, the total number of cells in a given population does not necessarily correlate directly with DA concentration, as certain species or strains may produce higher or lower levels of DA regardless of their population density. These variations illustrate the complexity of DA biosynthesis and suggest that several physiological and environmental factors influence DA production [43]. In line with the present study, Percopo et al. did not detect DA production in the strains of *P. allochrona* examined [44]. The strains of *P. mannii* also showed no toxin production, which is consistent with the results of previous studies [44,45,46,47]. In contrast to the present results, in which the strains of *P. calliantha*, *P. delicatissima*, *P. fraudulenta*, *P. galaxiae*, and *P. multistriata* did not produce detectable amounts of DA, several studies have reported DA production in these species under different environmental conditions and experimental setups (*P. calliantha* [32,34,47], *P. delicatissima* [31,32,33,48,49], *P. fraudulenta* [49,50,51], *P. galaxiae* [33,52], and *P. multistriata* [33,42,53]). These listed species are generally considered less toxic and do not necessarily produce DA under all conditions, which could explain the discrepancies between different studies. However, the evidence that these species have the potential for DA production under specific conditions suggests that some strains within these species could produce DA. These facts suggest that the toxicological potential of these species is variable and affected by specific biotic and abiotic factors.

A variety of factors could affect DA production in *Pseudo-nitzschia* species, including biological, physical, nutritional, and internal factors [1]. Biological factors, such as the presence of grazers (e.g., zooplankton), can stimulate or enhance DA production as a defence mechanism [54]. Physical factors, such as temperature, pH, photoperiod, salinity, and irradiance, have been shown to have a significant impact on DA synthesis, with several conditions favoring increased toxin production [8,55]. Nutritional factors, particularly the availability or limitation of key nutrients, such as silicates (Si), phosphorus (P), copper (Cu), iron (Fe), and ammonium (NH_4_^+^), play a crucial role in DA production, with nutrient stress often leading to increased toxin levels [1,56]. In addition, internal factors, including the growth phase and cell culture age, have been described to influence DA synthesis [57,58]. In this study, the focus was on standard cell culture conditions to evaluate DA production under controlled laboratory conditions. However, future research will examine the effects of different environmental parameters on toxin production to better understand the ecological and physiological triggers of DA synthesis in *Pseudo-nitzschia* species.

## 4. Conclusions

This study addressed some existing gaps regarding the genus *Pseudo-nitzschia* in the Central Adriatic Sea, specifically its growth rate, abundance, and toxin production across different growth phases. Out of eight analysed *Pseudo-nitzschia* species, only *P. pseudodelicatissima* was confirmed to produce domoic acid (DA). This species exhibited DA production when grown at 18 °C. Some strains of *P. pseudodelicatissima* showed continuous toxin production throughout their growth phases. In addition, nine out of eleven strains produced toxins in the decay phase. The highest cell density was found in *P. delicatissima* during the stationary phase, and the highest growth rates were recorded for *P. delicatissima*, *P. mannii,* and *P. pseudodelicatissima.* This research is of particular importance, as the genus *Pseudo-nitzschia* is present throughout all seasons in the Adriatic Sea and poses a potential threat to marine food webs and human health, especially in the context of shellfish aquaculture.

## 5. Materials and Methods

### 5.1. Cell Cultures

**Figure 5:**
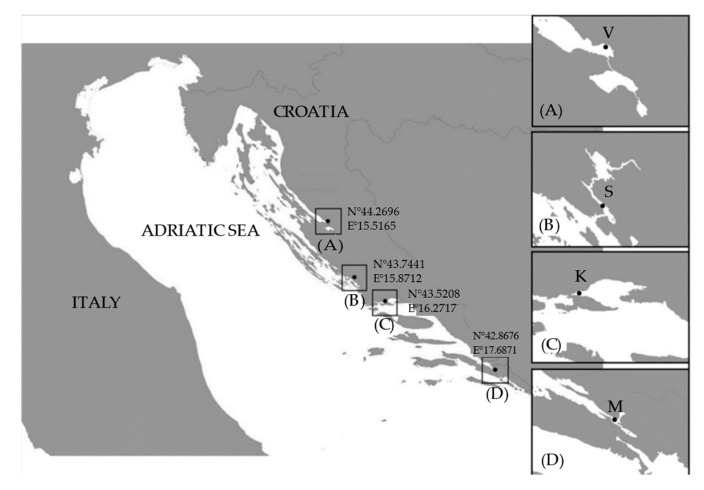
The study sites: (**A**) V—Velebit Channel, (**B**) S—Šibenik Channel, (**C**) K—Kaštela Bay, (**D**) M—Mali Ston Bay.

For this experiment, 54 monoclonal strains of *Pseudo-nitzschia* spp. were analysed, including eight species: *P. allochrona*, *P. calliantha*, *P. delicatissima*, *P. galaxiae*, *P. fraudulenta*, *P. mannii*, *P. multistriata*, *P. pseudodelicatissima*. Cell cultures were established by isolating single cells or chains with a glass micropipette under the Leica DMI 4000B (Leica Microsystems CMS, Wetzlar, Germany) inverted microscope from a net sample collected of four different aquaculture areas: Velebit Channel (44.2696° N, 15.5165° E), Šibenik Channel (43.7441° N, 15.8712° E), Kaštela Bay (43.5208° N, 16.2717° E), and Mali Ston Bay (42.8676° N, 17.6871° E) (Figure 5). After isolation, the cells were gradually rinsed in several drops of sterile medium on different microscope slides and then transferred into 48-well plates containing 1 mL of f/2 + Si cell medium. The species were determined using molecular methods. The species, strain names, isolation and experimental date, temperatures during the experiment, and GenBank numbers included in this study are listed in Table S1 in the Supplementary Materials. Cell cultures were grown in 500 mL or 1000 mL Erlenmeyer flasks containing up to 600 mL of sterilised f/2 medium enriched with silicates [59] within a temperature range of 16–19 °C (±0.5 °C) and 120 µmol photons m^−2^ s^−1^ (light: dark photoperiod of 12:12 h). The f/2 medium used was prepared with offshore seawater pre-filtered with GF 6 grade Whatman glass microfibers, stored in the dark at room temperature (20–24 °C) for at least two months and then autoclaved and filtered through a 0.22 µm membrane filter (mixed cellulose ester) after nutrient addition. The pH and salinity of the cell culture medium were monitored and adjusted to the appropriate ranges. The pH was maintained between 8.11 and 8.19, while the salinity was maintained at 35–36 PSU. The cell cultures were non-axenic.

### 5.2. Experimental Setup

In this study, growth and toxicity of all strains were examined in four growth phases: growth phase 1 (early exponential phase) in which the cells begin to divide and the growth rate begins to increase; growth phase 2 (late exponential phase) where cells divide rapidly and the growth rate reaches maximum; growth phase 3 (stationary phase) where growth rate slows down and cells number stays the same; growth phase 4 (decay phase) in which cells begin to die faster than they divide. In general, the cell cultures were examined within four months of isolation. The duration of the experiment was up to eighteen days: the cell cultures were examined under the light microscope and inoculated in the exponential phase (day 0). The daily growth of the *Pseudo-nitzschia* spp. cell cultures was monitored measuring in vivo fluorescence, using the sub-samples (1 mL) for flourescence measurements that were taken from each cell culture daily except on weekends, two hours after the start of the light phase, and each sample was measured three times and mean values were calculated for further analysis [60]. The correlation for each analysed *Pseudo-nitzschia* species between fluorescence and the corresponding cell abundance was determined by linear regression analysis. For all species, the slope was significant (*p* < 0.005) and R^2^ ranged from 0.6963 to 0.9761. For some species intercept was not significant (*p* ranged from 0.2121 to 0.8685) and was set to zero. (Figure S1). Fluorescence is a reliable indicator of the cell density throughout the exponential growth phase; however, the accuracy is not ensured in the decay phase. For this reason, the data corresponding to the decay phase were excluded from the analysis [61].

Cell cultures were homogenised almost daily by gentle manual stirring. *Pseudo-nitzschia* spp. cultures that produced DA were grown in triplicate. Samples were taken from each cell culture daily, except on weekends, to perform in vivo fluorescence measurements using a Turner Designs fluorometer (model 7200-000, Turner Designs, San Jose, CA, USA) to monitor the daily growth of the *Pseudo-nitzschia* spp. cell cultures; measurements were taken two hours after the start of the light phase, each sample was measured three times, and mean values were calculated for further analysis [60,61].

Cell culture sub-samples for toxin analysis and determination of the cell abundance were taken in four different growth phases, with additional sub-samples taken on the inoculation day for determination of cell abundance.

### 5.3. Cell Abundance and Growth Rate Determination

Sub-samples (3 mL) were taken from each cell culture to determine the cell density and fixed with acidic Lugol’s solution. At least 1000 cells and 50 fields were counted in a Sedgewick–Rafter chamber at 200× magnification with an inverted light microscope (Olympus CKX53, Olympus Corporation, Tokyo, Japan). Only viable cells were counted [62].

The cell abundance for toxic strains that were grown in triplicate was counted in one sub-sample from each growth phase. The cell counts for the two remaining replicates, which were not counted under the microscope, were calculated using the ratio between the cell count for the one counted replica and RFU apiece for each replica. Prior to sampling, cell cultures were homogenised manually. Cell cultures of *Pseudo-nitzschia* spp. that had a high cell density were diluted with filtered seawater to obtain approximately up to 30 cells per counting field. The specific growth rate (μ) was calculated from the data obtained from the fluorescence measurements, using the following formula:

$$\mu =\frac{\ln(f_{t2}/f_{t1})}{t_{2}-t_{1}}$$

The natural logarithm of f_t2_ characterises the fluorescence measured at time t_2,_ and f_t1_ characterises the fluorescence measured at time *t*_1_ [17,20]. To determine when the exponential phase begins and ends, semi-logarithmic plots of the logarithm of the measured fluorescence versus time were generated for each strain.

### 5.4. Toxin Analysis

For domoic acid analysis, up to 100 mL of the cell culture of *Pseudo-nitzschia* spp. was filtered through GF/F filters (Whatman, Maidstone, UK, pore size 0.7 µm). The cell culture was filtered using the lowest possible vacuum to prevent the cells from rupturing. In addition to the cell culture samples, we also filtered control samples of cell-free medium, specifically sterile f/2 medium with silicates, which served as a blank for comparative analysis and to identify any metabolites inherently present in the medium itself. The filters were frozen at −20 °C until analysis by liquid chromatography coupled with tandem mass spectrometry (LC–MS/MS). The filters containing the cells were placed in a 2 mL tube together with 1 mL of 100% methanol and 0.5 mm zirconia/glass beads. DA was extracted by mechanical disruption in a MagNA Lyser (Roche, Basel, Switzeralnd) at 6000 rpm for 30 s. Samples were then centrifuged twice at 14,000 rpm for 15 min at 4 °C, and after each centrifugation, the supernatant was collected in a new tube and filtered through a 0.22 µm membrane (Nylon Syringe Filter, 13 mm diameter, FilterBio, Nantong, China) prior to LC–MS/MS analysis. The filtered methanol extracts were analysed by LCMS/MS (Agilent Technologies, Santa Clara, CA, USA) to determine the DA content. The tandem mass spectrometer was equipped with a Triple Quad 6410, a Degasser 1200, a Quaternary Pump 1200, an Auto sampler 1290, and a Thermostated Column Compartment 1290. The chromatograph conditions for the Poroshell 120 (EC-C18, 2.1 mm × 50 mm, 2.7 μm) column coupled to the Poroshell 120 (EC-C18, 2.1 mm × 5 mm, 2.7 μm) pre-column were: flow 0.3 mL/min, temperature 30 °C, and mobile phase gradients are shown in Table 3. Mobile phase A consisted of 100% water with 2 mM ammonium formate and 50 mL formic acid, while mobile phase B consisted of 95% acetonitrile, 5% water with 2 mM ammonium formate, and 50 mM formic acid.

The multiple reaction mode (MRM) was performed in positive ion mode to quantify DA. For DA, electrospray ionisation (ESI) was used as the optimum ion source interface. The identification of DA was based on the retention time of DA in HPLC and the exact of protonated parent ion (312.2 *m*/*z*) and the most intense product ion (266.1 *m*/*z*) at collision energy 12 V. For qualitative identification, a second selected fragment (248.0 *m*/*z*) based on intensity is required at a collision energy of 12 V.

The quantification of DA was based on the calibration curves of six working standard solutions. To obtain these working standard solutions, the stock solution of DA (National Research Council of Canada, Halifax, NS, Canada) was diluted. The DA concentration in the stock solution was 3000 ng mL^−1^ and was prepared in methanol. The stock solution was diluted to six working solutions with the following concentrations: 30, 60, 90, 180, 300 and 450 ng mL^−1^. The sample volume injected into the LC–MS/MS was 2 µL. The retention time of DA was 2.60 min. The limit of detection (LOD) was based on a signal-to-noise ratio of 3:1. The LOD for DA was 11.025 ng mL^−1^. For this experiment, only the LOD was considered as the experiment was performed in a relatively small volume of cell culture medium.

The cellular DA concentration (ng DA cell ^−1^) was determined using the following formula: $DA\frac{ng}{cell}=\frac{\mathrm{DA}\;\mathrm{concentration}\left(\mathrm{ng}{\mathrm{mL}}^{-1}\right)\times \mathrm{volume}\;\mathrm{of}\;\mathrm{extraction}\;\mathrm{solution}(\mathrm{mL})}{\mathrm{cell}\;\mathrm{number}\left(cell{mL}^{-1}\right)\times \mathrm{volume}\;\mathrm{of}\;\mathrm{filtered}\;\mathrm{culture}(\mathrm{mL})}$

### 5.5. Statistics

Data analyses were performed using Excel^®^ (Microsoft^®^ Corp., Redmond, WA, USA) and RStudio (version 4.4.2, Boston, MA, USA) [63], respectively.

## Figures and Tables

**Table 1:** Growth rates and particulate domoic acid concentrations (pDA) of analysed *Pseudo-nitzschia* strains.

Species	Strain	pDA in Cell Culture (ng/mL)	Growth Rate µ (day^−1^)	Maximum Abundance × 10^3^ (cell mL^−1^)
*P. allochrona*	S222al	ND	0.26	322.20
	S223al	ND	0.83	301.20
	S280al	ND	1.18	289.40
*P. calliantha*	M074ca	ND	0.63	201.20
	V061ca	ND	0.83	276.68
	V065ca	ND	0.51	124.44
	V068ca	ND	0.63	277.60
	V070ca	ND	1.04	259.40
	V071ca	ND	0.46	270.00
	V072ca	ND	0.40	202.80
	V077ca	ND	0.52	200.60
	V079ca	ND	0.63	113.75
*P. delicatissima*	K057de	ND	0.53	512.22
	K058de	ND	0.65	789.60
	K129de	ND	0.64	636.60
	K134de	ND	0.66	515.80
	M075de	ND	0.74	482.00
	M085de	ND	0.96	411.20
	M088de	ND	0.48	509.20
	M144de	ND	0.33	566.40
	M461de	ND	1.66	641.60
	M462de	ND	1.52	514.40
	V040de	ND	0.64	808.60
	V041de	ND	0.55	819.00
	V042de	ND	0.60	612.40
	V043de	ND	0.53	677.40
*P. fraudulenta*	K450fr	ND	0.98	201.30
	K455fr	ND	0.78	117.20
	K456fr	ND	0.62	77.10
	K458fr	ND	1.04	102.20
*P. galaxiae*	K136ga	ND	1.09	466.80
	M232ga	ND	0.73	382.40
*P. mannii*	K231ma	ND	0.66	108.70
	K237ma	ND	0.37	130.80
	M233ma	ND	0.38	114.70
	M236ma	ND	1.01	105.30
	M239ma	ND	1.19	164.35
	M240ma	ND	1.19	130.80
	M241ma	ND	0.40	113.20
	V229ma	ND	1.10	119.64
*P. multistriata*	S290mu	ND	0.93	179.20
	S442mu	ND	0.98	100.60
*P. pseudodelicatissima*	K328ps	1.78–2.10	1.38	113.10
	K336ps	5.42–6.32	1.17	309.20
	K339ps	0.96–5.66	0.87	259.80
	K340ps	4.72	0.90	268.60
	K349ps	4.78	0.94	294.40
	K350ps	ND	0.68	96.27
	K351ps	5.44	0.92	278.00
	K352ps	0.96–6.26	0.67	293.40
	K356ps	0.22–0.93	0.78	236.40
	K357ps	1.00–4.01	0.88	187.96
	K358ps	0.68–3.93	0.94	277.80
	K359ps	0.28–0.68	1.13	237.60

ND, not detected.

**Table 2:** Mean cell abundances (cell mL^−1^) of replicates in growth phases, in which strains of *P. pseudodelicatissima* showed DA production, with mean domoic acid concentration in the cell culture and per cell (early exponential phase -> growth phase 1, exponential phase -> growth phase 2, stationary phase -> growth phase 3 and decay phase -> growth phase 4).

Strain	Growth Phase	Mean Abundance × 10^3^ (cell mL^−1^)	Mean DA Concentration in Culture (ng mL^−1^)	Mean DA Concentration (pg cell^−1^)
K328ps	3	89.20	1.78	0.0128
	4	105.44	2.10	0.0097
K336ps	4	316.33	6.32	0.0014
	5	271.83	5.42	0.0027
K339ps	2	48.51	0.96	0.0163
	3	209.55	4.18	0.0095
	4	282.60	5.66	0.0070
	5	202.16	4.04	0.0026
K340ps	5	236.03	4.72	0.0034
K349ps	5	238.62	4.78	0.0116
K351ps	5	272.54	5.44	0.0021
K352ps	2	48.79	0.96	0.0086
	3	281.37	5.62	0.0044
	4	313.56	6.26	0.0044
	5	223.43	4.46	0.0052
K356ps	3	233.23	0.78	0.0035
	4	222.42	0.22	0.0010
	5	154.35	0.93	0.0061
K357ps	2	89.83	1.00	0.0112
	3	187.17	3.89	0.0208
	4	165.07	4.01	0.0241
	5	140.90	3.53	0.0250
K358ps	2	69.42	1.09	0.0160
	3	196.40	3.93	0.0200
	4	286.95	2.98	0.0104
	5	92.45	0.68	0.0007
K359ps	2	79.61	0.28	0.0036
	3	247.47	0.54	0.0022
	4	224.36	0.34	0.0015

**Table 3:** Gradient conditions of mobile phases A and B for LC–MS/MS analysis of domoic acid.

Time (min)	A (%)	B (%)
0	90	10
4	20	80
6	20	80
6.5	90	10
10.5	90	10
11	90	10

## Data Availability

The original contributions presented in this study are included in the article/Supplementary Materials. Further inquiries can be directed to the corresponding author(s).

## Supplementary Materials

The following supporting information can be downloaded at: https://www.mdpi.com/article/10.3390/toxins17060307/s1, Table S1: S-Analysed cell cultures with species, strain IDs, dates, temperatures and GenBank numbers (majority of sequences are published in Bonačić et al. 2025 [29]). Figure S1: The correlation for each analysed *Pseudo-nitzschia* species between fluorescence and the corresponding cell abundance (cell mL^−1^). (A) *P. allochrona*; (B) *P. calliantha*; (C) *P. delicatissima*; (D) *P. fraudulenta*; (E) *P. galaxiae*; (F) *P. mannii*; (G) *P. multistriata*; (H) *P. pseudodelicatissima*. The scale is different for some species due to different fluorescence.
