# Supplementary material for: Growth Dynamics and Toxin Production of Pseudo-nitzschia Species Isolated from the Central Adriatic Sea
Source: Toxins (Basel). 2025 Jun 17;17(6):307. doi: 10.3390/toxins17060307 (PMC12197538; doi:10.3390/toxins17060307)
Supplement: Supplementary file 1 [file toxins-17-00307-s001.zip › toxins-3671098-supplementary.pdf]

Growth Dynamics and Toxin Production of *Pseudo-nitzschia* Species Isolated from the Central Adriatic Sea

Tina Tomašević, Jasna Arapov, Ivana Ujević, Tina Bonačić, Mia Bužančić, Antonija Bulić, Sanda Skejić, Romana Roje-Busatto and Živana Ninčević Gladan

Table S1. Analysed cell cultures with species, strain IDs, dates, temperatures and GenBank numbers (majority of sequences are published in Bonačić et al. 2025 [1]).

Species	Strain ID	Area	Isolation date	Start and end of the experiment	Temperature during experiment (°C)	LSU accession number (GenBank)	ITS accession number (GenBank)	rbcL accession number (GenBank)
P. allochrona	S222al	Šibenik	Sep 2022	28.10.-14.11.2022	19	PP216012	PP109422	PP213366
P. allochrona	S223al	Šibenik	Sep 2022	17.10.-31.10.2022	19	PP216013	PP109423	PP213367
P. allochrona	S280al	Šibenik	Oct 2022	28.11.-13.12.2022	19	PP216014	PP109425	PP213368
P. calliantha	M074ca	Mali Ston Bay	Feb 2022	22.4.-11.5.2022	18	OQ309026	OQ309033	
P. calliantha	V061ca	Velebit Bay	Feb 2022	22.4.-11.5.2022	18	PV367403		
P. calliantha	V065ca	Velebit Bay	Feb 2022	29.4.-11.5.2022	18	PV367404		
P. calliantha	V068ca	Velebit Bay	Feb 2022	22.4.-11.5.2022	18	PQ063246	PP109445	
P. calliantha	V070ca	Velebit Bay	Feb 2022	22.4.-11.5.2022	18	PQ002445	PP109446	PP213385
P. calliantha	V071ca	Velebit Bay	Feb 2022	22.4.-11.5.2022	18	PQ063247	PP109447	
P. calliantha	V072ca	Velebit Bay	Feb 2022	29.4.-11.5.2022	18	PQ063248	PP109448	
P. calliantha	V077ca	Velebit Bay	Feb 2022	22.4.-11.5.2022	18	PQ002442	PP109429	PP213386
P. calliantha	V079ca	Velebit Bay	Feb 2022	22.4.-11.5.2022	18	OQ309024	OQ309034	PP213387
P. delicatissima	K057de	Kaštela Bay	Dec 2021	4.4.-19.4.2022	18	OQ309088	OQ309040	PP213391
P. delicatissima	K058de	Kaštela Bay	Dec 2022	4.4.-19.4.2022	18	OQ309098	OQ309042	PP213329
P. delicatissima	K129de	Kaštela Bay	Mar 2022	17.5.-1.6.2022	18	OQ309091	OQ309044	PP213330
P. delicatissima	K134de	Kaštela Bay	Apr 2022	17.5.-1.6.2022	18	OQ309092	OQ309045	PP213337
P. delicatissima	M075de	Mali Ston Bay	Feb 2022	4.4.-19.4.2022	18	OQ309095	OQ309048	PP213349
P. delicatissima	M085de	Mali Ston Bay	Feb 2023	4.4.-19.4.2022	18	OQ309090	OQ309043	PP213351
P. delicatissima	M088de	Mali Ston Bay	Feb 2024	4.4.-19.4.2022	18	OQ309097	OQ309050	PQ366018
P. delicatissima	M144de	Mali Ston Bay	Apr 2022	28.10.-14.11.2022	19	OQ309089	OQ309041	PQ366020
P. delicatissima	M461de	Mali Ston Bay	Jan 2024	06.3.-15.3.2024	16	PQ002450	PQ002614	PQ351380
P. delicatissima	M462de	Mali Ston Bay	Jan 2024	06.3.-15.3.2024	16	PQ063251	PQ373931	
P. delicatissima	V040de	Velebit Bay	Nov 2021	18.3-4.4..2022	18	OQ309087	OQ309038	PP213381
P. delicatissima	V041de	Velebit Bay	Nov 2021	18.3-4.4..2022	18	OQ309086	OQ309039	PP213382
P. delicatissima	V042de	Velebit Bay	Nov 2021	18.3-4.4..2022	18	OQ309099	OQ309051	PP213383

P. delicatissima	V043de	Velebit Bay	Nov 2021	18.3-4.4..2022	18		OQ309085	OQ309052	PP213384
P. fraudulenta	K450fr	Kaštela Bay	Dec 2023	7.3.-18.3.2024	16		PQ002451	PQ002615	PQ351367
P. fraudulenta	K455fr	Kaštela Bay	Dec 2023	7.3.-18.3.2024	16		PQ002452	PQ002616	PQ351371
P. fraudulenta	K456fr	Kaštela Bay	Dec 2023	7.3.-18.3.2024	16		PQ002453	PQ002617	PQ351372
P. fraudulenta	K458fr	Kaštela Bay	Dec 2023	7.3.-18.3.2024	16		PQ002454	PQ002618	PQ351373
P. galaxiae	K136ga	Kaštela Bay	Apr 2022	17.5.-1.6.2022	18		OQ312051	OQ309100	PP213338
P. galaxiae	M232ga	Mali Ston Bay	Sep 2022	28.10-14.11..2022	19		OQ983983	OQ983978	PP213353
P. mannii	K231ma	Kaštela Bay	Sep 2022	17.10.-31.10.2022	19		PQ002457	PP109453	PQ366023
P. mannii	K237ma	Kaštela Bay	Sep 2022	28.10.-14.11.2022	19		OQ319216	OQ309150	PQ366024
P. mannii	M233ma	Mali Ston Bay	Sep 2022	28.10.-14.11.2022	19		PP215991	PP109454	PQ366028
P. mannii	M236ma	Mali Ston Bay	Sep 2022	17.10.-31.10.2022	19			PV357199	
P. mannii	M239ma	Mali Ston Bay	Sep 2022	28.11.-13.12.2022	19		OQ319217	OQ309151	
P. mannii	M240ma	Mali Ston Bay	Sep 2022	17.10.-31.10.2022	19		PP215993	PP109456	PQ366029
P. mannii	M241ma	Mali Ston Bay	Sep 2022	28.10.-14.11.2022	19		PP215994	PP109457	
P. mannii	V229ma	Velebit Bay	Sep 2022	17.10.-31.10.2022	19		PV367405		
P. multistriata	S290mu	Šibenik	Nov 2022	17.03.-3.4.2023	18		PP216017	PP109438	PP213370
P. multistriata	S442mu	Šibenik	Nov 2022	6.3.-15.3.2024	16		PQ002458	PQ002620	PQ351386
P. pseudodelicatissima	K328pa	Kaštela Bay	Nov 2022	23.1.-8.2.2023	18		PP216024	PP109439	PQ351357
P. pseudodelicatissima	K336ps	Kaštela Bay	Nov 2022	23.1.-8.2.2023	18		PP215948	PP109440	PP213343
P. pseudodelicatissima	K339ps	Kaštela Bay	Nov 2022	23.1.-8.2.2023	18		PQ063255		PQ366034
P. pseudodelicatissima	K340ps	Kaštela Bay	Nov 2022	17.2.-7.3.2023	18		PP215949	PP109470	
P. pseudodelicatissima	K349ps	Kaštela Bay	Nov 2022	17.2.-7.3.2023	18		PP215951	PP109441	PP213345
P. pseudodelicatissima	K350ps	Kaštela Bay	Nov 2022	17.2.-7.3.2023	18		PP215952	PP109442	PP213346
P. pseudodelicatissima	K351ps	Kaštela Bay	Nov 2022	17.2.-7.3.2023	18		PP215953	PP109477	PQ351359
P. pseudodelicatissima	K352ps	Kaštela Bay	Nov 2022	17.2.-7.3.2023	18		PP215954	PP109471	PQ351360
P. pseudodelicatissima	K356ps	Kaštela Bay	Dec 2022	17.3.-3.4.2023	18		PP215955	PP109472	PQ351361
P. pseudodelicatissima	K357ps	Kaštela Bay	Dec 2022	17.3.-3.4.2023	18		PP215956	PP109473	PQ351362
P. pseudodelicatissima	K358ps	Kaštela Bay	Dec 2022	17.3.-3.4.2023	18		PP215957	PP109474	PQ351363
P. pseudodelicatissima	K359ps	Kaštela Bay	Dec 2022	17.3.-3.4.2023	18		PP215958	PP109475	PQ351364

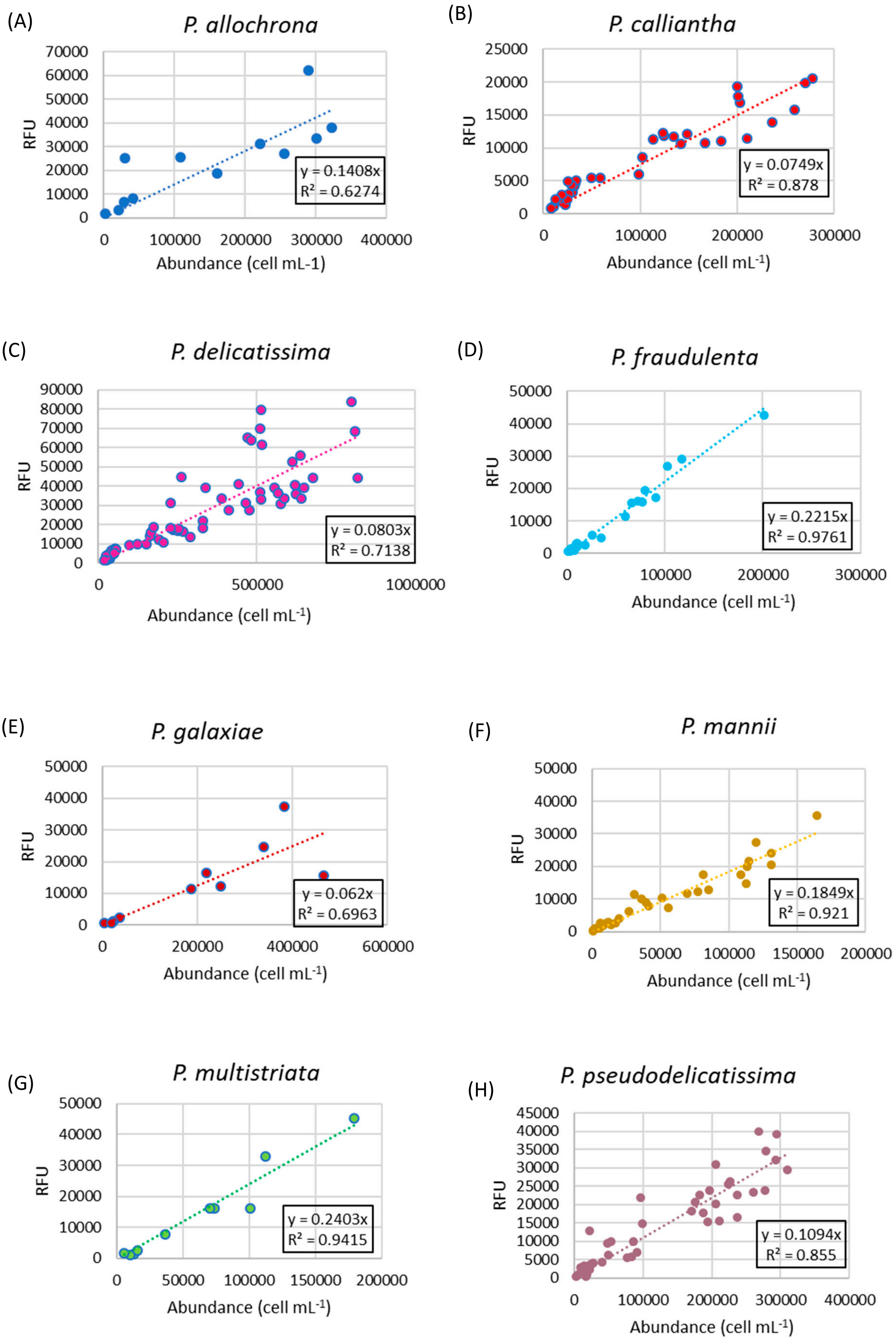

Figure S1. The correlation for each analysed *Pseudo-nitzschia* species between fluorescence and the corresponding cell abundance. (A) *P. allochrona*; (B) *P. calliantha*; (C) *P. delicatissima*; (D) *P. fraudulenta*; (E) *P. galaxiae* ;(F) *P. mannii*; (G) *P. multistriata*; (H) *P. pseudodelicatissima*. The scale is different for some species due to different fluorescence.

[1] T. Bonačić *et al.*, "Advancing the Taxonomy of the Diatom Pseudo-nitzschia Through an Integrative Study Conducted in the Central and Southeastern Adriatic Sea," *Plants*, vol. 14, no. 2, 2025, doi: 10.3390/plants14020245.
